# Cemented compared to uncemented femoral stems in total hip replacement for displaced femoral neck fractures in the elderly: study protocol for a single-blinded, randomized controlled trial (CHANCE-trial)

**DOI:** 10.1186/s12891-016-1253-y

**Published:** 2016-09-20

**Authors:** Ghazi Chammout, Olle Muren, Henrik Bodén, Mats Salemyr, Olof Sköldenberg

**Affiliations:** Karolinska Institute, Department of Clinical Sciences at Danderyd hospital, Stockholm, S-182 88 Sweden

**Keywords:** Total hip arthroplasty, Femoral neck fracture, Femoral stem, Randomized controlled trial, Outcome

## Abstract

**Background:**

Total hip replacement (THR) is the preferred method for the active and lucid elderly patient with a displaced femoral neck fracture (FNF). But controversy still exists regarding using cemented or uncemented stem in these patients. The aim of this study is to compare a cemented and uncemented femoral stem in patients 65–79 years treated surgically with THR for displaced FNF.

**Methods/design:**

In a single-centre, single-blinded, randomized controlled trial, we will include 140 patients aged 65-79 years with an acute displaced FNF and randomize them in a 1:1 ratio to a cemented tapered or a uncemented tapered hydroxyapatite - coated femoral stem. A cemented cup will be used in both groups. The patients will be blinded for allocation. The primary endpoints will be the incidence of all hip-related complications and health-related quality of life evaluated with EuroQol-5D (EQ-5D) index up to 2 years after surgery. Secondary outcomes will be overall mortality, general medical complications and hip function. The follow-up will be at 3 months, 1 and 2 years. Further follow-ups after end of study will be at 4 and 10 years. Results will be analysed using 95 % CI’s for the effect size. A regression model will also be used to adjust for stratification factor.

**Discussion:**

The ethical committee at Karolinska Institutet has approved the study. An interim analysis on the primary endpoints will be performed when half the sample size is included. The results from the study will be disseminated to the medical community via presentations and publications in relevant medical journals. The study will provide evidence if a cemented or uncemented femoral stem is preferable in THR for elderly patients with a displaced FNF.

**Trial registration:**

The trial is registered at clinicaltrials.gov (NCT02247791), October 21, 2013.

## Background

Total hip replacement (THR) is the preferred method for the active and lucid elderly patient with a displaced femoral neck fracture (FNF) [1, 2]. Comparisons between cemented and uncemented stems in hip arthroplasty for patients with a FNF have almost consistently favoured cemented fixation, mainly because of greater deterioration in pain, walking ability, use of walking aids and activity of daily living [3] and because of a higher incidence of hip related complication such as periprosthetic fracture [4] for uncemented implants. Despite this, recent reports on modern, hydroxyapatite-coated femoral stems used for this patient-group have shown promising early results [5-7]. In addition, bone cement implantation syndrome (BCIS), is more prevalent in cemented than in uncemented stems [8]. It is also a commonly occurring phenomenon in patients treated for FNF with cemented arthroplasty and severe BCIS has a significant impact on early and late mortality [9]. Thus, the use of uncemented stems for this patient group may still be justified.

We hypothesized that an uncemented, proximally porous and hydroxyapatite coated femoral stem used in THR for a displaced FNF would not be associated with more adverse peri- and postoperative hip-related complications compared with a THR using cemented stem, and that the health-related quality of life for the patients would be equivalent at 2 years.

## Methods/design

### Trial design, settings and location

This single centre, single-blinded, prospective randomized controlled trial will be performed between 2009 and 2025 at the Orthopaedic department Danderyd Hospital, Stockholm, Sweden. Danderyd Hospital is an emergency regional teaching hospital affiliated with the Karolinska Institute and has a catchment area of approximately 500,000 inhabitants. The guidelines of Good Clinical Practise (ICH-GCP) will be followed [10]. The Local Ethics Committee at Karolinska Institute approved the protocol. The guidelines of the CONSORT Statement will be followed [11] for the final paper and the SPIRIT guidelines for the study protocol [12].

### Randomization and blinding

The patients will be block-randomized in a 1:1 ratio, to receive either cemented or uncemented stem. We will use sealed envelopes and randomization will be stratified by sex to ensure that the sex distribution will be the same in both groups. The study subjects will be blinded to choice of treatment. To verify that the blinding is maintained during the study, the patients will be asked if they knew their assigned allocation arm at the 2-year follow-up. The surgeons and staff are not blinded during the study.

### Study subjects and eligibility criteria

All patients with a displaced FNF who are admitted to Danderyd Hospital will be screened for participation in the study. Research nurses who identify eligible patients before surgery screen all patients at arrival to the hospital. The first author (G.C.) or one of the other co-authors will include patients and acquire informed consent. All who agree to participate and give their oral and written informed consent will be included if they fulfil the inclusion criteria. The inclusion criteria are an acute (within 48 h) displaced FNF (Garden III to IV) [13] after a low-energy trauma (i.e. fall), an age 65–79 years, no concurrent joint disease or previous fracture in the lower extremities, an intact cognitive function (no diagnosis of dementia and at least seven correct answers on a ten-item Short Portable Mental Status Questionnaire [14], ability to ambulate independently with or without the help of walking aids. The age limits for THR (65–79 years) are standard in our hospital and in most Swedish hospitals. We will exclude patients with pathological fractures, and those with rheumatoid arthritis or symptomatic osteoarthritis of the hip. We will also exclude those who, because of severe co-morbidities, are deemed not suitable for a THR by the anaesthesiologist, and those who are unsuitable to participate in the study for any other reason (substance abuse, alcoholism). Based on our previous experience in randomized clinical trials in this population, [1, 5] we expect to include and receive informed consent from approximately half of the target population (i.e. 65–79 years and fulfilling other inclusion criteria).

The baseline data of the patients’ health-related quality of life and hip function will be obtained retrospectively for the last week.

### Surgical intervention

Surgery in both groups will be performed by a consultant or a specialist experienced in both procedures using a direct lateral approach [15] with the patient in the lateral decubitus position. Preoperative planning will be performed using digital software (MDesk; RSA Biomedical AB, Umeå, Sweden). The modular CPT (Zimmer, Warsaw, Indiana, USA) collarless, polished, tapered femoral component manufactured from cobalt-chromium will be used used in the cemented group. The Bi-Metric stem (Biomet, Warsaw, Indiana, USA) is a tapered, proximally coated (plasma-sprayed, commercially pure [CP]) titanium femoral stem and will be used in the uncemented group. A 32 mm cobalt-chromium will be used for all patients.

In the acetabular component we will use a cemented XLPE cup and a vacuum-mixed low-viscosity cement with gentamicin. All surgeons have a long experience with all implants so no learning curve is expected. Low-molecular-weight heparin (Fragmin, Pfizer, Täby, Sweden) postoperative day 1 and for at least 10 days postoperatively will be given as thromboprophylaxis. Antibiotic prophylaxis with Cloxacillin 2 g (Ekvacillin; Meda Sweden) will be given preoperatively, followed by two additional doses during the first 24 h. Patients in both groups will be mobilised with full weight bearing with the aid of two crutches as tolerated and to abandon crutches at their own convenience. After 6 weeks they will be permitted to mobilise without further restriction. A physiotherapist will follow all patients for the first 3 months after surgery.

### Outcome measurements

#### Primary endpoints

The primary endpoints will be 1) the incidence of all hip-related complications up to 2 years after surgery and 2) change in health-related quality of life assessed with EQ-5D index (EuroQol) [16] up to 2 years. Hip-related complications are defined as intra- and postoperative periprosthetic fractures, dislocation, wound infection both superficial and deep, loosening both early and late and revision of any prosthetic implant for any reason.

#### Secondary endpoints

The secondary endpoints include overall mortality as well as hip function evaluated with Harris hip score (HHS) [17]. The score is widely used for evaluating hip function after THA and has also been validated as a self-reported instrument and for patients with fractures of the femoral neck [18–20]. Other endpoints includes pain when walking in the involved hip (measured with a visual analogue scale (VAS) [21] and activities of daily living (ADL) [22]. Other data collected include intraoperative bleeding, duration of surgery and vital signs (blood pressure, heart rate and pulse oximetry before, during and after stem insertion) to estimate any decrease in value during cementing. In addition, we measure serological markers of inflammation (Interleukin-6 [IL-6], C-reactive protein [CRP]) and thrombosis (D-dimer) at operative day, post-operative day (POD) 1, POD 4 and at 3 months. We will record all general medical complications including cardiovascular events and thromboembolism.

#### Radiology

Radiological analysis includes the presence of radiolucent lines around the stem and cup in the zones of Gruen et al. [23] and Delee and Charnley [24]. Any circumferential radiolucent lines around the implants will be defined as loosening. Heterotopic ossification is graded according to the classification of Brooker et al. [25]. All radiological evaluations will be done by an independent radiologist not otherwise involved in the study.

### Data collection and follow-ups

The primary assessment will establish that the patient fulfils all inclusion/exclusion criteria and identify any comorbidity. The patients will then be interviewed by a research nurse regarding living conditions, mobility, activities of daily living (ADL) [22], status and health-related quality of life according to the EQ-5D during the last week before the fracture as a baseline. For all primary and secondary endpoints including ADL the patients themselves will be providing data during the study period. It is obvious that the patients’ ability to record this correctly while awaiting urgent surgery may be questioned. It is, however, impossible to collect these data in a prospective manner and the method is regularly used in patients with a fracture of the hip [1, 2, 19]. Patients who are unable or unwilling to attend follow-ups will be interviewed by telephone or they will send their answers by mail. We will use the unique Swedish personal id-number and collect data prospectively throughout the study period through a combination of a search of our databases, follow-ups and the Swedish Hip Arthroplasty Register. Non hip-related adverse events (AEs) and serious adverse events (SAEs) will be collected throughout the study period (2 years). Although the primary endpoints will be evaluated at 2 years, the study will also include a 4 and 10-year follow-up. The study visits are presented in Table 1.

**Table 1:** All visits in the study

	d 0	d 0	0	+1 d	+4 d	5d	3 m.	1 y	2 y	4 y	10 y
	Screening	Inclusion	Randomization and surgery	POD1	POD4	End of In-pt stay	Follow-up	Follow-up	Follow-up	Follow-up	Follow-up
Screening for eligibility	x										
Informed consent		x									
Baseline data		x									
Randomization			x								
X-ray	x			x				x	x	x	x
HHS		x						x	x	x	x
VAS		x						x	x	x	x
EQ-5D		x						x	x	x	x
Adverse events			x	x		x	x	x	x	x	x
Intraoperative			x								
Blood test		x		x	x		x				

X-ray = anterioposterior and lateral radiographs, HHS = Harris hip score, EQ-5D = Quality of life score, Intraoperative = Intraoperative measurements including blood pressure, puls rate, pulse oxyometry before, during and after femoral stem insertion, VAS = Visual Analogue Scale for hip pain, Bloodtest = C-reactive protein (CRP), IL-6, Hb, D-dimer

### Data quality assurance

The monitoring of the study and the data quality assurance will be identical to the methods published in two other study protocols from our research group [26, 27]. Briefly, we will use an external monitor to ensure that ICH- GCP [10] and all aspects of the protocol are followed. All study data will be collected and managed in a digital case report form (CRF) using REDCap electronic data capture tools hosted at Karolinska Institutet [28]. Each subject will receive a unique identification number, which will be linked to the CRF. The data will then be blinded correspondingly in all data analyses.

### Sample size and power analysis

Prior to the study start, a sample size calculation was done. To show non-inferiority with 80 % power of the primary endpoint all hip-related complications between the two groups, assuming a total complication rate of 20 %, and with a non-inferiority limit of 15 % requires 60 patients in each group. The assumed 20 % complication rate includes also minor hip-related complications as per previously published papers ranging between 10 and 20 % [1, 2, 6]. To show non-inferiority with 80 % power of the primary variable health related quality of life – HRQoL, as measured with EQ-5D requires 40 patients in each group, and with a non-inferiority limit of 0.1, assuming a value of 0.73 (SD 0.18) 1 year after the surgery [5]. The alpha (2-tailed) is set at 0.025 since we have two endpoints for the power calculation. Since this patient group has a 1-year mortality of 10 %, 70 patients in each group should be sufficient for the study.

### Analysis

Analyses of outcome are based on the intention-to-treat principle and all patients remain in their randomized group regardless of any further surgical intervention. Patients with missing data (i.e. EQ-5D, HHS VAS etc.) at any of the follow-ups are analysed with the last observation carried forward (imputed). Descriptive statistics (means and standard deviations) will be used to describe the patient characteristics and outcome variables at the measurement points. Fisher’s exact test will be used to test the primary endpoint. We will use the Student’s *t*-test and Levene’s test for comparison of the functional outcomes with 95 % CI presented. We will use Kaplan-Meier survival curves with log-rank test for patient and hip re-operation survival analysis. The analyses will be performed with SPSS 22.0 for Mac (SPSS, Chicago, Illinois) statistical software.

## Discussion

The strengths of the study are the patient blinding and the study design with clinically relevant outcome as the primary endpoint. The ethical committee at Karolinska Institutet has approved the study. An interim analysis on the primary endpoints will be performed when half the sample sizes in included. If there is a disproportionate number of hip-related, or other, complications in the uncemented group the study will be stopped. The results from the study will be disseminated to the medical community via presentations and publications in relevant medical journals. We believe the internal validity of this trial is good due to the strict inclusion criteria, rigorous follow-up and the blinding of the patients. Broad exclusion criterias can affect the external validity and generalizability of a study but they are mainly focusing on excluding patients with malignant disease or those with contraindications for either treatment methods as well as patients with cognitive impairment.

## Conclusion

The present trial will provide evidence for the future choice of stem fixation for hip arthroplasty in elderly patients below 80 years of age with a displaced femoral neck fracture and without cognitive impairment.

## Abbreviations

ADL: Activities of daily living
AE: Adverse event
BCIS: Bone cement implantation syndrome
CI: Confidence interval
CONSORT: Consolidated standards of reporting trials
CRF: Case report form
CRP: C-reactive protein
EQ-5D: A standardised instrument for use as a measure of health outcome
EuroQoL: European quality of life scale
FNF: Femoral neck fracture
HHS: Harris hip score
ICH-GCP: International conference on harmonisation-good clinical practice
IL-6: Interleukin-6
POD: Post-operative day
REDCap: Research electronic data capture
SAE: Serious adverse event
SPIRIT: Standard protocol Items: recommendations for interventional trials
THR: Total hip replacement
VAS: Visual analogue scale
XLPE: Highly cross-linked polyethylene
